# Health Resorts

**Published:** 1902-10-18

**Authors:** 


### The Institutional Workshop.

### HEALTH RESORTS.

### SCARBOROUGH.

In days gone by Scarborough consisted of everything that was fashionable, and still Scarborough is a dressy place although the advent of the tripper and the increasing popularity of the town as a place of residence have led to considerable changes in the character of its clientèle. As a health resort, however, Scarborough while retaining most of its old advantages, offers now many more attractions than it did some years ago, for it must be confessed that there have been periods in its history when the sanitary arrangements were far from being all that man could desire. No doubt even now there are parts which are capable of much improvement, but take the town as a whole it may now be regarded as well up to, and in the newer portions distinctly above, the average of sea-side places. In the winter months Scarborough is distinctly cold, the easterly winds are very trying, and in stormy weather none but the strong can get much out of doors, but from soon after Easter to about November it is a very useful bracing tonic place, which may be prescribed in many conditions of debility, and in the more advanced stages of convalescence from acute disease.

Old Scarborough is an ancient town, having evidently been a place of some importance in Saxon days, and having been fought for many times. The present castle, the ruins of which form so picturesque a centre to every view of the bay, was commenced in the middle of the twelfth century, but it had been a fortified place long before that, so that one need hardly be surprised to find some traces in the older portions of the town of the odours of antiquity. The old port of Scarborough, with its piers and docks, nestling under the castle, has, however, become almost lost in the midst of the large modern and well-built town which has grown up all around, and the Scarborough that we know to-day dates practically from the middle of the last century, when the Spa grounds were so laid out as to furnish a pleasing and convenient meeting ground and promenade for all the fashionable people who assembled there; for curiously enough Scarborough is still a spa at which chalybeate waters, equal in strength to some more famous, are still drunk for the sake of their tonic properties. These waters contain carbonate of iron, and sulphates of lime and magnesia. They are slightly tonic and very slightly aperient, and they possess this great virtue, that they deteriorate when kept, so that they should be taken as they flow fresh from the spring. Those who will get up in the morning and drink the waters at the Spa will find that they gain in many ways besides the mere drinking of iron and magnesia. Scarborough is a large and busy town of between 30,000 and 40,000 inhabitants. There is always something going on; even in winter it is not absolutely dull, while in summer it is definitely gay. Moreover, in the higher portions of the town it is always bracing, so that without having any very special virtues it is a health resort of much utility.

### MALVERN.

The Malvern Hills consist of a single chain of peaks, some nine miles in extent from north to south, lying between the extensive plain which extends far away on the Worcestershire side, and the more or less undulating country over which, on the Hereford side, the eye of the observer may wander even to the Welsh mountains. The hills are formed of a central core of eruptive rocks, by the intrusion of which the strata on each side have been considerably disturbed, and the *débris* of which forms the soil of all the higher parts of Malvern. These causes, no doubt, conduce much to the rapid drying of the soil after rain, which is one of the special boasts of the place. Dryness of soil and dryness of air are, indeed, two of the features to which much of its health-giving power is traceable.

Associated with the same causes, and more particularly with the absence of those oxidisable substances which are commonly found in damp and stagnant air, is the considerable amount of ozone which the atmosphere of Malvern generally contains; for although one may well question the direct curative effects of ozone, its presence does at least testify to the freshness and purity of, and to the absence of decomposable material in, the air in which it is found.

Malvern is a small town snugly placed on the eastern side of the Malvern Hills, sufficiently elevated above the extensive plain which lies below to partake of the bracing effects of the upper strata of the air, while well protected by the hills themselves from the more tempestuous winds. So far as the sanitary concerns of the town are concerned, it may be stated that while its water-supply is good its position gives every facility for drainage, and that the council possesses an isolation hospital for the reception of infectious cases. Of these, however, there seems to be but a small supply, if we may judge from the zymotic death-rate, the average of which for eight years is given as 0·46 per 1,000 living. Indeed, the total death-rate is very low, the average for the 13 years ending with 1897 having been only 12·2, including the deaths of visitors.

The town possesses many of the usual attributes of a well-patronised and almost fashionable watering-place. It has a club, assembly-rooms, music, public gardens, cricket, football, hockey, tennis, and, that modern essential to happiness, "golf," the latter in great excellence. It is a great centre also for excursions, a large number of places of interest being within driving distance.

The great attraction of Malvern, however, consists of the hills, with their great grassy slopes, with well-kept walks in every direction, with plenty of seats, with carriage roads over the more easy gradients, with fresh breezes from whichever quarter the wind may blow, and with such an expanse of view as can certainly be obtained from no other point in England, a view including on a clear day a sight of sixteen counties of England and Wales—at least so it is said.

The cases which receive benefit at Malvern are of many kinds. Probably, so far as the climate itself is concerned, the primary fact to be taken into consideration is its beneficial influence upon anæmia, for we can hardly doubt that in many of the cases of gout, phthisis, dyspepsia and nervous collapse which flock to Malvern, the influence of the climate upon the accompanying blood condition is an important factor in the results obtained. But for many years Malvern has been an important hydropathic centre, with the result that the range of cases which may usefully be sent there is considerably wider than merely climatic considerations would suggest.

Thus we find anæmia, nervous break-down, dyspepsia, "feeble circulation," or "deficient vitality," often another name for anæmia, various kidney troubles, chronic heart diseases (sometimes spoken of as "fatty heart," but often, no doubt, cases of gradually advancing cardiac dilatation), and many cases of phthisis, all doing well under the combined effects of hydropathy, fresh air, and graduated exercises.

Naturally the main season is in the summer and autumn, when both invalids and pleasure-seekers "make hay while the sun shines." But every year it is being seen that the the injuriousness of an English winter does not depend entirely upon the cold, but rather upon the damp and fogs, and especially the town fogs; and that if an invalid can but obtain dryness and sunshine and purity of air he may get through the winter fairly well. These are the very things that Malvern offers, and we need not wonder that at Malvern, as at other dry and sunny health resorts, a very considerable number of visitors stay through the winter, or indeed go there especially for the sake of escaping the evils associated with the winter season in smoky towns or in low-lying districts.

